# Case report: A case of Acute Macular Neuroretinopathy secondary to Influenza A virus during Long COVID

**DOI:** 10.3389/fimmu.2023.1302504

**Published:** 2024-01-15

**Authors:** Jiaqi Zhang, Yihao Xia, Xiaodong Li, Runxi He, Xuejun Xie

**Affiliations:** ^1^ Chengdu University of Traditional Chinese Medicine, Chengdu, China; ^2^ Eye School of Chengdu University of Traditional Chinese Medicine, Chengdu, China; ^3^ The First Affiliated Hospital, Guizhou University of Traditional Chinese Medicine, Guiyang, China; ^4^ Hospital of Chengdu University of Traditional Chinese Medicine, Chengdu, China

**Keywords:** retinoic acid depletion syndrome, neutrophil extracellular trap, inflammation, virus infection, microvascular endothelial dysfunction, retinal diseases

## Abstract

Ocular abnormalities have been reported in association with viral infections, including Long COVID, a debilitating illness caused by the Severe Acute Respiratory Syndrome Coronavirus 2 (SARS-CoV-2). This report presents a case of a female patient diagnosed with Acute Macular Neuroretinopathy (AMN) following an Influenza A virus infection during Long COVID who experienced severe inflammation symptoms and ocular complications. We hypothesize that the rare occurrence of AMN in this patient could be associated with the immune storm secondary to the viral infection during Long COVID.

## Highlights

- A case of AMN secondary to Influenza A during Long COVID is reported.
- Influenza A infection was confirmed by antibody detection of a nasopharyngeal swab.
- The initial optical coherence tomography image of the fundus examination showed macular edema in the AMN patient.

## Introduction

Acute Macular Neuroretinopathy (AMN) is a rare condition that was first described by Bos and Deutman, and it is characterized (1) by subacute paracentral scotoma and damage to the outer retinal layers. It is widely agreed among scholars that AMN has been associated with various vascular factors (2), viral infections (3), and the use of oral contraceptives (1). With the outbreak of Coronavirus Disease 2019 (COVID-19), an increasing number of patients have experienced diverse symptoms (4) as adverse outcomes following Severe Acute Respiratory Syndrome Coronavirus 2 (SARS-CoV-2) infection (5). Interestingly, the incidence (6) of AMN has significantly risen following the COVID-19 outbreak. Although the precise mechanism of retinal diseases caused by SARS-CoV-2 remains unknown, several scholars have proposed hypotheses for infection pathogenesis. These hypotheses suggest that SARS-CoV-2 targets cells, enhances binding to the Angiotensin-Converting Enzyme 2 (ACE2) receptor, triggers the release of pro-inflammatory factors, causes vascular endothelial dysfunction (7), and eventually leads to a systemic cellular immune storm (8–10). Notably, ACE2 receptors are expressed in retinal structures (6). Influenza virus infection is a common cause of respiratory tract diseases in humans. Moreover, severe Influenza A Viruses (IAVs) infection can lead to the production of a large number of chemokines and inflammatory factors, resulting in a severe immune storm (11). Additionally, unusual cases of secondary infections with violent immune inflammation caused by influenza viruses post SARS-CoV-2 infection warrant further attention.

## Case description

**Figure 1:**
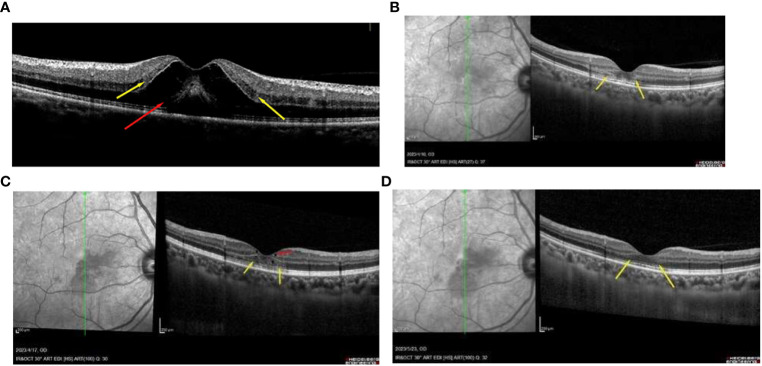
**(A)** OCT image of the patient with the paracentral scotoma with oral 3-day oseltamivir, showing the macular edema (red arrowhead) and the hyperreflectivity at the retinal outer layer (yellow arrowhead). **(B)** the AMN lesions in the outer retinal layer can be found easily (yellow arrowhead) with 1 week of oral oseltamivir. **(C)** some repair of the microcapsule cavity (red arrowhead) in the retina and the structure of the outer retinal layer 2 weeks of treatment (yellow arrowhead). **(D)** the retinal structure was obviously repaired at the 1-month follow-up appointment (yellow arrowhead).

**Figure 2:**
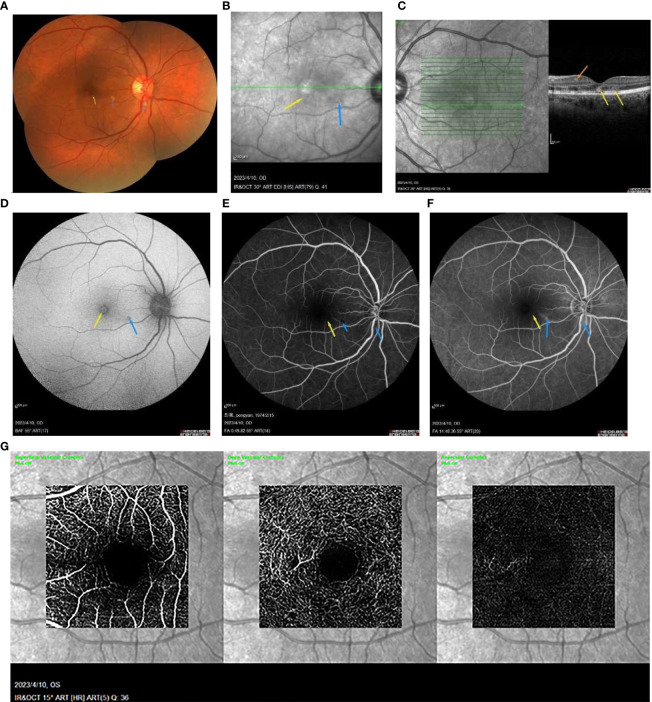
**(A)** the wedge-like AMN lesion is classic (yellow arrowhead) and some perivascular cotton plaques (blue arrowhead) in the fundus photograph. **(B)** IR image showing a wedge-like AMN lesion (yellow arrowhead) and radial weak reflex lesions (blue arrowhead). **(C)** the SD-OCT image shows the hyperreflectivity at the retinal outer layer (yellow arrowhead) with PAMM lesion in inner retinal layers (orange arrowhead). **(D)** the AMN lesion is an unremarkable weak reflex lesion (yellow arrowhead), and there is a perivascular weak reflex lesion not detectable (blue arrowhead) in the AF image. **(E, F)** FFA showed the unremarkable weak reflex lesions (yellow arrowhead) and the perivascular occluded fluorescence (blue arrowhead) at the early stage, then the weak reflex lesion in macula (yellow arrowhead), and the strong perivascular fluorescence at the later stage (blue arrowhead). **(G)** the OCTA image showed the decreased density of SCP and DCP.

A 49-year-old Chinese woman was admitted to our hospital, presenting with a 3-day history of paracentral scotoma in both eyes. An Influenza A virus infection was confirmed through antibody detection from a nasopharyngeal swab. She reported experiencing a viral prodrome 1 week prior to the onset of visual symptoms, which included hyperpyrexia. Laboratory examinations revealed elevated levels of white blood cells, neutrophil percentage, hypersensitive C-reactive protein, and procalcitonin, indicating severe systemic inflammation. The patient had completed a full course of the COVID-19 vaccine and had not received the influenza vaccine. She had been suffering from fatigue and memory loss since she contracted SARS-CoV-2 3 months prior. Before seeking medical attention, she had taken oral oseltamivir for 5 days. The best-corrected visual acuities in both eyes were 20/50, with slit-lamp examinations revealing no abnormalities. China CDC has reported that all the random gene samples collected in China between December 2022 and January 2023 were identified as Omicron strains. Optical coherence tomography (OCT) conducted at a local hospital revealed macular edema (ME) in both eyes (**Figure 1A**). Classic wedge-like lesions were detected in infrared reflectance (IR) and fundus photography at our hospital (**Figures 2A, B**), but weak reflex lesions were unremarkable in Autofluorescence (AF) (**Figure 2D**). Spectral-domain optical coherence tomography (SD-OCT) indicated hyperreflectivity at the retinal outer layer (**Figure 1B**), which is consistent with paracentral acute middle maculopathy (PAMM) (**Figure 2C**). Further examinations using fundus fluorescein angiography (FFA) and optical coherence tomography angiography (OCTA) were conducted. FFA results showed preretinal arteriolar obstruction in both eyes, with perivascular fluorescence shielded in the early angiography, and perivascular fluorescence leakage and mild telangiectasia in the later stage, corresponding to the lesions in the IR image (**Figures 2E, F**). OCTA revealed an incomplete macular arch ring structure and hypoperfusion in both the superficial capillary plexus (SCP) and the deep capillary plexus (DCP) (**Figure 2G**). The patient was diagnosed with AMN secondary to Influenza A. Following treatments of oral Oseltamivir, glucocorticoid anti-inflammatories, and traditional Chinese medicine, the patient reported a slight persistent scotoma. A 1-month follow-up showed the presence of a microcapsule cavity in the retina of both eyes (**Figure 1C**) and significant repair of the retinal structure (**Figure 1D**).

## Discussion

Numerous studies have demonstrated that female patients (12–14) are more susceptible to complications associated with Long COVID (LC). This susceptibility could potentially be attributed to factors such as active centromeric features (15), variations in sex hormone levels (16), persistent deficiencies in dendritic cells, and dysbiosis (12). However, further investigation is required to ascertain the significance of these differences. Recent research has further uncovered that residual SARS-CoV-2 can persist in various organ systems (17–20), thereby increasing oxidative stress response (21) and cytotoxic effects (7), which may contribute to LC development. The prevalence of LC is estimated to exceed 10% (22, 23), with some reports suggesting that it could impact 50–70% (24, 25) of hospitalized patients. Neurological manifestations (26) of LC encompass fatigue (27, 28) and memory (29) impairment. Studies have also disclosed long-term alterations in the rigidity of blood cells and vessels (30), potentially affecting oxygen transport and reducing blood flow density in LC patients during follow-up appointments (26). Additionally, as COVID-19 prevention and control measures are relaxed (31), the incidence of influenza infection has rebounded to pre-pandemic levels (32). Influenza virus infection may cause a delayed innate immune response (33). This, in turn, may disrupt the biphasic immune response, triggering virus-independent mechanisms (34, 35) following immune escape. These mechanisms, including immune dysregulation and vascular inflammation, could potentially exacerbate LC symptoms.

Intriguingly, research has indicated that both SARS-CoV-2 and all three subtypes (36) of IAVs can activate the Retinoic Acid-Inducible Gene I (RIG-I) pathway (37, 38), leading to the formation of Neutrophil Extracellular Traps (NETs) (39). This suggests a potential similarity between the two viruses. RIG-I receptors, found in various cells such as dendritic cells (40), retinal Müller cells (41), and endothelial cells (42), play a pivotal role in retinal inflammation and the production of Type I Interferon (IFN) immune responses by retinal pigment epithelium (RPE) (43). Notably, several studies have identified a potential link between IAVs and eye disorders (44–46). Moreover, IAVs have been found to replicate in RPE cells (45, 46). Furthermore, it has been observed that LC can potentially impair the function and structure of dendritic cells (47), leading to adaptive immune response disorders that exacerbate immune disorders and microcirculatory vascular dysfunction (48). Some researchers propose that SARS-CoV-2 infection can disrupt the peripheral immune system, leading to the release of cytokines and resulting in long-term chronic low-grade inflammation (14), oxidative stress (49), and tissue damage (39). Studies on primary care patients (50) with COVID-19 have found that neutrophil levels, which are chronically affected by LC, participate in the cytokine storm through activated inflammatory vesicles (51). The persistence of NETs in LC suggests a potential risk of long-term imbalance of the innate immune response and low levels of the prothrombotic state (39, 52). The activation of neutrophils indicates their involvement in immune thrombosis, while NETs play a crucial role in the mechanism of thrombosis (53).

In the context of the innate immune response, the RIG-I receptor, a Pattern Recognition Receptor (PRR), plays a crucial role in recognizing viral infections (54). RIG-I is responsible for the recognition of viral RNA through the ubiquitination (55) of long non-coding RNAs (lncRNAs), aerobic glycolysis, and phosphorylation. This recognition triggers a cascade reaction between RIG-I/Melanoma Differentiation-Associated Protein 5 (MDA5) and Mitochondrial Antiviral Signaling Protein (MAVS), promoting the production of IFN and coordinating the antiviral immune response (56–58). Additionally, RIG-I is responsible for the secretion of inflammatory factors and cytokines such as Interleukin-6 (IL-6), Interleukin-8 (IL-8), and Vascular Endothelial Growth Factor (VEGF) (59). However, the regulation of the immune response by RIG-I is not stable, necessitating further studies to clarify the mechanisms involved.

The RIG-I pathway of the innate immune system primarily initiates the signaling cascade during the early stage of viral infection (60). However, severe infections and hyperthermia can cause a rapid depletion of retinol and a reduction in retinoic acid levels (61, 62). Consequently, the RIG-I pathway becomes blunted and eventually collapses due to this depletion (63). This disruption (62) in the production of Type I IFN results in the immune defense mechanism transitioning into adaptive immunity, leading to the activation of a large number of centrocytes. This activation triggers the generation of cytokine storms through the over-discharge pathway of the nuclear factor kappa B (NF-κ B) (64), causing a condition known as “Retinoic Acid Depletion Syndrome” (RADS) (64). RADS leads to vascular dysfunction, tissue damage, and the generation of reactive oxygen species (ROS) (65). Therefore, we hypothesize that the low-grade inflammatory state induced by LC, coupled with the hyperthermia caused by acute IAV infection, led to RADS and the activation of NETs. This activation, in turn, stimulated the release of proinflammatory factors, leading to retinal vascular endothelial dysfunction. This ultimately resulted in hyperreflectivity and severe ME in the outer structures of the retina. However, further studies are needed to confirm the link between the relevant pathological mechanisms and the severe AMN and ME in this patient.

Given the limited research on AMN, our study may be the first to report a case of AMN secondary to IAV during LC. Notably, our research differs from previous reports (44, 66–68) as we observed early signs of binocular macular edema and disorganization of the retinal layer following the resolution of ME. The etiology of AMN is complex and remains to be fully established. Turbeville et al. (3) have postulated that vascular factors may unify infection, inflammation, and ischemia in the pathogenesis of this condition. Our reported case may be associated with the microvascular mechanism of AMN. A study (69) conducted by Marc et al. revealed that infections can trigger innate immune responses, which in turn can cause cytotoxicity, dysfunctional angiogenesis, and ME. Furthermore, research (70) on ME secondary to other retinal diseases has indicated that swelling of Müller cells and activated neuroglia can lead to a decrease in Platelet-Derived Growth Factor (PGDF) and the release of pro-inflammatory cytokines such as VEGF through leukocyte-mediated processes. This occurs when the human body is in a state of ischemia or inflammation, and can induce impaired blood-retinal barrier, increased vascular permeability, dysfunctional angiogenesis, accumulation of subretinal fluid, and formation of retinal edema. In fact, similar to Gomel et al. (71), we strongly believe that the possible mechanisms for the ME of the patient may include systemic immune storm such as hyperpyrexia and an increasing index of inflammation. In our case, the patient who had a history of SARS-CoV-2 infection accompanied by long-term fatigue and memory loss. Combined with the positive influenza A test, we suspect that the patient’s AMN with PAMM was likely secondary to IAV infection during LC.

PAMM refers to banded hyperreflective lesions (72) in the inner layer of the retina that manifest clinically. Several reports have suggested that PAMM may occur earlier than lesions such as in the Foveal Avascular Zone (FAZ) (73), and it could be an early manifestation of retinal vascular diseases. Multiple studies have revealed that the occurrence of PAMM indicates ischemia (72) of the deep retinal capillaries and impairment of the deep capillary complex, which is likely the main site of progression of retinal ischemia. Influenza virus can cause disturbances in redox and impairments such as anoxia (74, 75), which may explain why the patient experienced retinal inner layer injury, PAMM, and decreased density of SCP and DCP. Additionally, the occurrence of retinal ischemia and PAMM may have prompted the retinal inner layer to develop tiny gaps, which could have been caused by increased levels of extracellular glutamate and inflammation in the retina due to swelling Müller cells (76). Finally, the tiny gaps subsided as the inflammation disappeared and the patient’s systemic symptoms improved.

In conclusion, the occurrence of AMN in a patient who had taken oral oseltamivir during the early stages of an Influenza A infection could potentially be attributed to the excessive viral load in the body and the ensuing severe inflammatory reaction. Following a short-term oral corticosteroid treatment, the patient’s symptoms were alleviated and the retinal structure restored. However, due to the absence of follow-up in the later stages of this patient’s treatment, there is a relative lack of observation of the disease’s prognosis. Furthermore, the sample size of this study is small, indicating the need for more data to analyze the clinical impact and mechanism of AMN caused by influenza virus infection during LC.

## Data availability statement

The original contributions presented in the study are included in the article/supplementary material. Further inquiries can be directed to the corresponding author.

## Ethics statement

The studies involving humans were approved by Ethical Medical Committee of Hospital of Chengdu University of traditional Chinese Medicine. The studies were conducted in accordance with the local legislation and institutional requirements. The participants provided their written informed consent to participate in this study. Written informed consent was obtained from the individual(s) for the publication of any potentially identifiable images or data included in this article.

## Author contributions

JZ: Writing – original draft, Writing – review & editing. YX: Investigation, Supervision, Writing – review & editing. XL: Investigation, Supervision, Writing – review & editing. RH: Investigation, Writing – review & editing. XX: Writing – review & editing, Writing – original draft.

## References

[1] Bos PJM Deutman AF. Acute macular neuroretinopathy. Am J Ophthalmol (1975) 80:573–84. doi: 10.1016/0002-9394(75)90387-6 1180301

[2] Bhavsar KV Lin S Rahimy E Joseph A Freund KB Sarraf D. Acute macular neuroretinopathy: A comprehensive review of the literature. Survey Ophthalmol (2016) 61:538–65. doi: 10.1016/j.survophthal.2016.03.003 26973287

[3] Turbeville SD Cowan LD Gass JDM. Acute macular neuroretinopathy: a review of the literature. Surv Ophthalmol (2003) 48(1):1–11. doi: 10.1016/s0039-6257(02)00398-3 12559324

[4] Xie Y Xu E Bowe B Al-Aly Z. Long-term cardiovascular outcomes of COVID-19. Nat Med (2022) 28:583–90. doi: 10.1038/s41591-022-01689-3 PMC8938267 35132265

[5] Davis HE Assaf GS McCorkell L Wei H Low RJ Re’em Y. Characterizing long COVID in an international cohort: 7 months of symptoms and their impact. eClinicalMedicine (2021) 38:101019. doi: 10.1016/j.eclinm.2021.101019 34308300 PMC8280690

[6] Azar G Bonnin S Vasseur V Faure C Salviat F Clermont CV. Did the COVID-19 pandemic increase the incidence of acute macular neuroretinopathy? J Clin Med (2021) 10:5038. doi: 10.3390/jcm10215038 34768555 PMC8585041

[7] Proal AD VanElzakker MB. Long COVID or post-acute sequelae of COVID-19 (PASC): an overview of biological factors that may contribute to persistent symptoms. Front Microbiol (2021) 12:698169. doi: 10.3389/fmicb.2021.698169 34248921 PMC8260991

[8] Sheth JU Narayanan R Goyal J Goyal V. Retinal vein occlusion in COVID-19: A novel entity. Indian J Ophthalmol (2020) 68:2291–3. doi: 10.4103/ijo.IJO_2380_20 PMC7727974 32971697

[9] Wiersinga WJ Rhodes A Cheng AC Peacock SJ Prescott HC. Pathophysiology, transmission, diagnosis, and treatment of coronavirus disease 2019 (COVID-19): A review. JAMA (2020) 324:782. doi: 10.1001/jama.2020.12839 32648899

[10] Ali MAM Spinler SA. COVID-19 and thrombosis: From bench to bedside. Trends Cardiovasc Med (2021) 31:143–60. doi: 10.1016/j.tcm.2020.12.004 PMC7836332 33338635

[11] Meineke R Rimmelzwaan G Elbahesh H. Influenza virus infections and cellular kinases. Viruses (2019) 11:171. doi: 10.3390/v11020171 30791550 PMC6410056

[12] Zhang H Sun Y Wang Y Yazici D Azkur D Ogulur I. Recent developments in the immunopathology of COVID -19. Allergy (2023) 78:369–88. doi: 10.1111/all.15593 PMC10108124 36420736

[13] Torjesen I. Covid-19: Middle aged women face greater risk of debilitating long term symptoms. BMJ (2021) 372:n829. doi: 10.1136/bmj.n829 33766927

[14] Maamar M Artime A Pariente E Fierro P Ruiz Y Gutiérrez S. Post-COVID-19 syndrome, low-grade inflammation and inflammatory markers: a cross-sectional study. Curr Med Res Opin (2022) 38:901–9. doi: 10.1080/03007995.2022.2042991 PMC8935459 35166141

[15] Gupta S Nakabo S Blanco LP O’Neil LJ Wigerblad G Goel RR. Sex differences in neutrophil biology modulate response to type I interferons and immunometabolism. Proc Natl Acad Sci USA (2020) 117:16481–91. doi: 10.1073/pnas.2003603117 PMC7368314 32601182

[16] Klein SL Marriott I Fish EN. Sex-based differences in immune function and responses to vaccination. Trans R Soc Trop Med Hygiene (2015) 109:9–15. doi: 10.1093/trstmh/tru167 PMC4447843 25573105

[17] Tejerina F Catalan P Rodriguez-Grande C Adan J Rodriguez-Gonzalez C Muñoz P. Post-COVID-19 syndrome. SARS-CoV-2 RNA detection in plasma, stool, and urine in patients with persistent symptoms after COVID-19. BMC Infect Dis (2022) 22:211. doi: 10.1186/s12879-022-07153-4 35240997 PMC8892394

[18] Goh D Lim JCT Fernaíndez SB Joseph CR Edwards SG Neo ZW. Corrigendum: Case report: Persistence of residual antigen and RNA of the SARS-CoV-2 virus in tissues of two patients with long COVID. Front Immunol (2022) 13:1036894. doi: 10.3389/fimmu.2022.1036894 36275692 PMC9583834

[19] Menuchin-Lasowski Y Schreiber A Lecanda A Mecate-Zambrano A Brunotte L Psathaki OE. SARS-CoV-2 infects and replicates in photoreceptor and retinal ganglion cells of human retinal organoids. Stem Cell Rep (2022) 17:789–803. doi: 10.1016/j.stemcr.2022.02.015 PMC8943915 35334213

[20] Cheung CCL Goh D Lim X Tien TZ Lim JCT Lee JN. Residual SARS-CoV-2 viral antigens detected in GI and hepatic tissues from five recovered patients with COVID-19. Gut (2022) 71:226–9. doi: 10.1136/gutjnl-2021-324280 34083386

[21] Corrao S Gervasi F Di Bernardo F Natoli G Raspanti M Catalano N. Immunological characteristics of non-intensive care hospitalized COVID-19 patients: A preliminary report. JCM (2021) 10:849. doi: 10.3390/jcm10040849 33669527 PMC7921979

[22] Davis HE McCorkell L Vogel JM Topol EJ. Long COVID: major findings, mechanisms and recommendations. Nat Rev Microbiol (2023) 21:133–46. doi: 10.1038/s41579-022-00846-2 PMC9839201 36639608

[23] Bull-Otterson L Baca S Saydah S Boehmer TK Adjei S Gray S. Post–COVID conditions among adult COVID-19 survivors aged 18–64 and ≥65 years — United States, March 2020–November 2021. MMWR Morb Mortal Wkly Rep (2022) 71:713–7. doi: 10.15585/mmwr.mm7121e1

[24] Martín-Sánchez FJ Martínez-Sellés M Molero García JM Moreno Guillén S Rodríguez-Artalejo F Ruiz-Galiana J. Insights for COVID-19 in 2023. Rev Esp Quimioter (2023) 36:114–24. doi: 10.37201/req/122.2022 PMC10066911 36510683

[25] Ceban F Ling S Lui LMW Lee Y Gill H Teopiz KM. Fatigue and cognitive impairment in Post-COVID-19 Syndrome: A systematic review and meta-analysis. Brain Behavior Immun (2022) 101:93–135. doi: 10.1016/j.bbi.2021.12.020 PMC8715665 34973396

[26] Osiaevi I Schulze A Evers G Harmening K Vink H Kümpers P. Persistent capillary rarefication in long COVID syndrome. Angiogenesis (2023) 26:53–61. doi: 10.1007/s10456-022-09850-9 35951203 PMC9366128

[27] Nalbandian A Sehgal K Gupta A Madhavan MV McGroder C Stevens JS. Post-acute COVID-19 syndrome. Nat Med (2021) 27:601–15. doi: 10.1038/s41591-021-01283-z PMC8893149 33753937

[28] Montani D Savale L Noel N Meyrignac O Colle R Gasnier M. Post-acute COVID-19 syndrome. Eur Respir Rev (2022) 31:210185. doi: 10.1183/16000617.0185-2021 35264409 PMC8924706

[29] Fernández-Castañeda A Lu P Geraghty AC Song E Lee M-H Wood J. Mild respiratory SARS-CoV-2 infection can cause multi-lineage cellular dysregulation and myelin loss in the brain. bioRxiv [Preprint] (2022). doi: 10.1101/2022.01.07.475453

[30] Kubánková M Hohberger B Hoffmanns J Fürst J Herrmann M Guck J. Physical phenotype of blood cells is altered in COVID-19. Biophys J (2021) 120:2838–47. doi: 10.1016/j.bpj.2021.05.025 PMC8169220 34087216

[31] Sovann LY Sar B Kab V Yann S Kinzer M Raftery P. (H3N2) virus outbreak in the Kingdom of Cambodia during the COVID-19 pandemic of 2020. Int J Infect Dis (2021) 103:352–7. doi: 10.1016/j.ijid.2020.11.178 PMC10290288 33249287

[32] Olsen SJ Winn AK Budd AP Prill MM Steel J Midgley CM. Changes in influenza and other respiratory virus activity during the COVID-19 pandemic—United States, 2020–2021. Am J Transplant (2021) 21:3481–6. doi: 10.1111/ajt.16049 PMC8653380 34624182

[33] Conenello GM Tisoncik JR Rosenzweig E Varga ZT Palese P Katze MG. A single N66S mutation in the PB1-F2 protein of influenza A virus increases virulence by inhibiting the early interferon response *in vivo* . J Virol (2011) 85:652–62. doi: 10.1128/JVI.01987-10 PMC3020033 21084483

[34] Dorward DA Russell CD Um IH Elshani M Armstrong SD Penrice-Randal R. Tissue-specific immunopathology in fatal COVID-19. Am J Respir Crit Care Med (2021) 203:192–201. doi: 10.1164/rccm.202008-3265OC 33217246 PMC7874430

[35] Mazzaglia G. Long COVID syndrome: lesson learned and future implications. JCM (2023) 12:3450. doi: 10.3390/jcm12103450 37240555 PMC10219445

[36] Huo C Jin Y Zou S Qi P Xiao J Tian H. Lethal influenza A virus preferentially activates TLR3 and triggers a severe inflammatory response. Virus Res (2018) 257:102–12. doi: 10.1016/j.virusres.2018.09.012 30248373

[37] Righetto I Gasparotto M Casalino L Vacca M Filippini F. Exogenous players in mitochondria-related CNS disorders: viral pathogens and unbalanced microbiota in the gut-brain axis. Biomolecules (2023) 13:169. doi: 10.3390/biom13010169 36671555 PMC9855674

[38] Guo XJ Thomas PG. New fronts emerge in the influenza cytokine storm. Semin Immunopathol (2017) 39:541–50. doi: 10.1007/s00281-017-0636-y PMC5580809 28555383

[39] Shafqat A Omer MH Albalkhi I Alabdul Razzak G Abdulkader H Abdul Rab S. Neutrophil extracellular traps and long COVID. Front Immunol (2023) 14:1254310. doi: 10.3389/fimmu.2023.1254310 37828990 PMC10565006

[40] Han L Zhuang M Deng J Zheng Y Zhang J Nan M. SARS-CoV-2 ORF9b antagonizes type I and III interferons by targeting multiple components of the RIG-I/MDA-5–MAVS, TLR3–TRIF, and cGAS–STING signaling pathways. J Med Virol (2021) 93:5376–89. doi: 10.1002/jmv.27050 PMC8242602 33913550

[41] Sauter MM Noel H Brandt CR. The RLR intrinsic antiviral system is expressed in neural retina and restricts lentiviral transduction of human Mueller cells. Exp Eye Res (2023) 236:109647. doi: 10.1016/j.exer.2023.109647 37689341 PMC10834037

[42] Xu S Jin T Weng J. Endothelial cells as a key cell type for innate immunity: A focused review on RIG-I signaling pathway. Front Immunol (2022) 13:951614. doi: 10.3389/fimmu.2022.951614 35865527 PMC9294349

[43] Schustak J Twarog M Wu X Wu HY Huang Q Bao Y. Mechanism of nucleic acid sensing in retinal pigment epithelium (RPE): RIG-I mediates type I interferon response in human RPE. J Immunol Res (2021) 2021:1–14. doi: 10.1155/2021/9975628 PMC8235977 34239945

[44] Ashfaq I Vrahimi M Waugh S Soomro T Grinton ME Browning AC. Acute macular neuroretinopathy associated with acute influenza virus infection. Ocular Immunol Inflammation (2021) 29:333–9. doi: 10.1080/09273948.2019.1681470 31697568

[45] Michaelis M Geiler J Klassert D Doerr HW Cinatl J. Infection of human retinal pigment epithelial cells with influenza A viruses. Invest Ophthalmol Vis Sci (2009) 50:5419. doi: 10.1167/iovs.09-3752 19553611

[46] Yoser SL Forster DJ Rao NA. Systemic viral infections and their retinal and choroidal manifestations. Survey Ophthalmol (1993) 37:313–52. doi: 10.1016/0039-6257(93)90064-E 8387231

[47] Galati D Zanotta S Capitelli L Bocchino M. A bird’s eye view on the role of dendritic cells in SARS-CoV-2 infection: Perspectives for immune-based vaccines. Allergy (2022) 77:100–10. doi: 10.1111/all.15004 PMC8441836 34245591

[48] Maggi E Azzarone BG Canonica GW Moretta L. What we know and still ignore on COVID-19 immune pathogenesis and a proposal based on the experience of allergic disorders. Allergy (2022) 77:1114–28. doi: 10.1111/all.15112 PMC8652765 34582050

[49] Jing H Wu X Xiang M Liu L Novakovic VA Shi J. Pathophysiological mechanisms of thrombosis in acute and long COVID-19. Front Immunol (2022) 13:992384. doi: 10.3389/fimmu.2022.992384 36466841 PMC9709252

[50] Jukema BN Smit K Hopman MTE Bongers CCWG Pelgrim TC Rijk MH. Neutrophil and eosinophil responses remain abnormal for several months in primary care patients with COVID-19 disease. Front Allergy (2022) 3:942699. doi: 10.3389/falgy.2022.942699 35966226 PMC9365032

[51] Aymonnier K Ng J Fredenburgh LE Zambrano-Vera K Münzer P Gutch S. Inflammasome activation in neutrophils of patients with severe COVID-19. Blood Adv (2022) 6:2001–13. doi: 10.1182/bloodadvances.2021005949 PMC8741335 34991159

[52] Pisareva E Badiou S Mihalovičová L Mirandola A Pastor B Kudriavtsev A. Persistence of neutrophil extracellular traps and anticardiolipin auto-antibodies in post-acute phase COVID-19 patients. J Med Virol (2023) 95:e28209. doi: 10.1002/jmv.28209 36226380 PMC9874393

[53] Yang J Wu Z Long Q Huang J Hong T Liu W. Insights into immunothrombosis: the interplay among neutrophil extracellular trap, von willebrand factor, and ADAMTS13. Front Immunol (2020) 11:610696. doi: 10.3389/fimmu.2020.610696 33343584 PMC7738460

[54] Kong F You H Zheng K Tang R Zheng C. The crosstalk between pattern-recognition receptor signaling and calcium signaling. Int J Biol Macromolecules (2021) 192:745–56. doi: 10.1016/j.ijbiomac.2021.10.014 34634335

[55] Liu J Ji Q Cheng F Chen D Geng T Huang Y. The lncRNAs involved in regulating the RIG-I signaling pathway. Front Cell Infect Microbiol (2022) 12:1041682. doi: 10.3389/fcimb.2022.1041682 36439216 PMC9682092

[56] Zheng Y Deng J Han L Zhuang M-W Xu Y Zhang J. SARS-CoV-2 NSP5 and N protein counteract the RIG-I signaling pathway by suppressing the formation of stress granules. Sig Transduct Target Ther (2022) 7:22. doi: 10.1038/s41392-022-00878-3 PMC8785035 35075101

[57] Zheng Y Zhuang M-W Han L Zhang J Nan M-L Zhan P. Severe acute respiratory syndrome coronavirus 2 (SARS-CoV-2) membrane (M) protein inhibits type I and III interferon production by targeting RIG-I/MDA-5 signaling. Sig Transduct Target Ther (2020) 5:299. doi: 10.1038/s41392-020-00438-7 PMC7768267 33372174

[58] Deng J Zheng Y Zheng S Nan M Han L Zhang J. SARS-CoV-2 NSP7 inhibits type I and III IFN production by targeting the RIG-I/MDA5, TRIF, and STING signaling pathways. J Med Virol (2023) 95:e28561. doi: 10.1002/jmv.28561 36755358

[59] Udawatte DJ Rothman AL. Viral suppression of RIPK1-mediated signaling. mBio (2021) 12:e01723–21. doi: 10.1128/mBio.01723-21 PMC8406217 34372694

[60] Hu Z van der Ploeg K Chakraborty S Arunachalam PS Mori DAM Jacobson KB. Early immune markers of clinical, virological, and immunological outcomes in patients with COVID-19: a multi-omics study. Elife (2022) 11:e77943. doi: 10.7554/eLife.77943 36239699 PMC9566856

[61] Chow KT Gale M Loo Y-M. RIG-I and other RNA sensors in antiviral immunity. Annu Rev Immunol (2018) 36:667–94. doi: 10.1146/annurev-immunol-042617-053309 29677479

[62] Sarohan AR Kızıl M İnkaya AÇ Mahmud S Akram M Cen O. A novel hypothesis for COVID-19 pathogenesis: Retinol depletion and retinoid signaling disorder. Cell Signalling (2021) 87:110121. doi: 10.1016/j.cellsig.2021.110121 34438017 PMC8380544

[63] Sirén J Imaizumi T Sarkar D Pietilä T Noah DL Lin R. Retinoic acid inducible gene-I and mda-5 are involved in influenza A virus-induced expression of antiviral cytokines. Microbes Infection (2006) 8:2013–20. doi: 10.1016/j.micinf.2006.02.028 16797201

[64] Sarohan AR. COVID-19: endogenous retinoic acid theory and retinoic acid depletion syndrome. Med Hypotheses (2020) 144:110250. doi: 10.1016/j.mehy.2020.110250 33254555 PMC7481114

[65] Huang Z Xu X Li J Gu L Yue Y Sun F. RIG-I contributes to dsDNA-induced innate immune activation in human brain microvascular endothelial cells. Mol Immunol (2022) 152:78–85. doi: 10.1016/j.molimm.2022.10.009 36306644

[66] Strzalkowski P Steinberg JS Dithmar S. COVID-19-assoziierte akute makuläre Neuroretinopathie [COVID-19-associated acute macular neuroretinopathy]. Ophthalmologie (2023) 120(7):767–70. doi: 10.1007/s00347-022-01704-5 35943530 PMC9361229

[67] Ahmed W Suri A Ahmed A. COVID-19 and Acute Macular Neuroretinopathy – An underlying association? Ann Med Surg (Lond) (2022) 78:103847. doi: 10.1016/j.amsu.2022.103847 35734676 PMC9207085

[68] David JA Fivgas GD. Acute macular neuroretinopathy associated with COVID-19 infection. Am J Ophthalmol Case Rep (2021) 24:101232. doi: 10.1016/j.ajoc.2021.101232 34778601 PMC8577875

[69] De Smet MD. Insights into the Physiopathology of Inflammatory Macular Edema, in: Developments in ophthalmology (2017). Available at: https://www.karger.com/Article/FullText/455279 (Accessed September 9, 2023). 10.1159/000455279 28351051

[70] Daruich A Matet A Moulin A Kowalczuk L Nicolas M Sellam A. Mechanisms of macular edema: Beyond the surface. Prog Retinal Eye Res (2018) 63:20–68. doi: 10.1016/j.preteyeres.2017.10.006 29126927

[71] GomelNShorRLippinNSegalOGreenbaumESchwartzS. COVID-19 pandemic lockdowns’ Impact on visual acuity of diabetic macular edema: A large cohort. Ophthalmologica (2023) 246:1–8. doi: 10.1159/000527942 36380651

[72] Maltsev DS Kulikov AN Burnasheva MA Chhablani J. Prevalence of resolved paracentral acute middle maculopathy lesions in fellow eyes of patients with unilateral retinal vein occlusion. Acta Ophthalmologica (2020) 98(1):e22–8. doi: 10.1111/aos.14196 31347293

[73] Burnasheva MA Maltsev DS Kulikov AN Sherbakova KA Barsukov AV. Association of chronic paracentral acute middle maculopathy lesions with hypertension. Ophthalmol Retina (2020) 4:504–9. doi: 10.1016/j.oret.2019.12.001 31948908

[74] Nencioni L Sgarbanti R Amatore D Checconi P Celestino I Limongi D. Intracellular redox signaling as therapeutic target for novel antiviral strategy. CPD (2011) 17:3898–904. doi: 10.2174/138161211798357728 21933147

[75] Amatore D Sgarbanti R Aquilano K Baldelli S Limongi D Civitelli L. Influenza virus replication in lung epithelial cells depends on redox-sensitive pathways activated by NOX4 -derived ROS . Cell Microbiol (2015) 17:131–45. doi: 10.1111/cmi.12343 PMC4311438 25154738

[76] Burggraaff MC Trieu J De Vries-Knoppert WAEJ Balk L Petzold A. The clinical spectrum of microcystic macular edema. Invest Ophthalmol Vis Sci (2014) 55:952. doi: 10.1167/iovs.13-12912 24398089

